# Severe COVID-19 in pediatric age: an update on the role of the anti-rheumatic agents

**DOI:** 10.1186/s12969-021-00559-5

**Published:** 2021-05-04

**Authors:** Giorgio Costagliola, Erika Spada, Rita Consolini

**Affiliations:** Section of Rheumatology and Clinical Immunology, Division of Pediatrics, Department of Clinical and Experimental Medicine, University of Pisa, Via Roma 67, 56126 Pisa, Italy

**Keywords:** Anakinra, Canakinumab, Coronavirus, Children, Macrophage activation syndrome, Multisystem inflammatory syndrome in children, SARS-CoV-2, Tocilizumab

## Abstract

**Background:**

SARS-CoV-2 can induce an immune impairment and dysregulation, finally resulting in the massive release of inflammatory mediators (cytokine storm), strongly contributing to the pulmonary and systemic manifestations in severe coronavirus disease 2019 (COVID-19). As a consequence, different drugs active on the immune system have been proposed for the treatment of the disease in adults.

**Role of the anti-rheumatic agents in children:**

Children are more likely to develop a mild disease course, as the severe form of COVID-19 is identified in less than 5% of the pediatric patients. Moreover, in children a peculiar disease phenotype, defined as multisystem inflammatory syndrome in children (MIS-C) is observed, representing the most severe expression of the inflammatory dysregulation caused by SARS-CoV-2. The limited experience with the severe pediatric COVID-19 and MIS-C does not allow conclusions about the role of the immune pharmacological approach, and therefore the treatment of these conditions represents a considerable clinical challenge. The use of chloroquine, hydroxychloroquine, and colchicine in the early disease stages is not sufficiently supported by evidence, and there is an increasing interest in the role of biologic agents, including anti-IL-1 and anti-IL-6 agents, in the prevention and treatment of the severe manifestations of COVID-19.

**Conclusion:**

The therapeutic approach to pediatric COVID-19 is multidisciplinary, and anti-rheumatic agents have a prominent role in severe disease. This paper reviews the rationale for the use of anti-rheumatic agents in pediatric COVID-19 and MIS-C and the clinical experience with the single drugs. Finally, the areas of potential improvement in the use of anti-rheumatic agents, including the optimization of the drug choice and the timing of administration, are discussed.

## Background

Coronavirus disease 2019 (COVID-19) is clinically characterized by a variable spectrum of disease severity, ranging from asymptomatic patients to cases featured by an upper respiratory infection, pneumonia and, potentially, acute respiratory distress syndrome (ARDS), septic shock, disseminated intravascular coagulation, and multi-organ failure (MOF) [1]. Disease severity appears to be higher among elderly patients with significant comorbidities (diabetes, hypertension, chronic cardiac, or pulmonary disorders) and smokers [2]. Children and infants with COVID-19 frequently have milder clinical symptoms and are less likely to develop severe disease [3, 4]. Indeed, while the incidence of severe disease requiring admission to an intensive care unit is estimated to be about 20% in the adult population [1], the admission rate to intensive care unit is 2–3% in pediatric patients, and fatal cases are rare (0,08%) [5, 6]. Although the pathogenesis of COVID-19 has not been fully elucidated, it is widely recognized that it is not primarily dependent on the cytopathic action of the virus and that the immune system plays a central role. During infection, an aberrant immune response can be elicited, resulting in the massive release of cytokines and chemokines (“cytokine storm”), which contributes to pulmonary and systemic tissue damage, leading to the clinical manifestations of severe COVID-19, including ARDS and MOF [7]. Moreover, children infected by SARS-COV-2 can develop, during the acute infection or in the following weeks, a peculiar clinical phenotype, defined as “multisystem inflammatory syndrome in children” (MIS-C). In this condition, considered the most severe clinical expression of pediatric SARS-CoV-2 infection, children show a severe systemic inflammatory picture (circulatory shock, hypotension, myocardial dysfunction), which can show common features with Kawasaki disease (KD) [8–10].

Although severe COVID-19 (with ARDS) and MIS-C are two markedly different clinical entities, current immunological and pathogenic knowledge suggest that in both conditions the cytokines interleukin-1 (IL-1) and IL-6 have a pivotal role in initiating and maintaining the inflammatory response. In the absence of a specific therapy, current treatment for patients with COVID-19 is based on a combination of broad-spectrum antiviral agents, anticoagulants, and anti-inflammatory drugs [11, 12]. The increasing understanding of the immunopathogenic mechanisms of the severe forms of COVID-19 has provided the opportunity for the use of drugs selectively targeting the immune response in adult patients [13], with demonstrated clinical benefits. The potential utility of an immune approach in children has still not been definitively determined because of the poor clinical practice experienced in patients with severe and critical COVID-19 disease. In this paper, we review the current knowledge on the use of anti-rheumatic agents in COVID-19 and MIS-C, and we discuss the main perspectives for the improvement of the therapeutic approach. In particular, we focus on the factors influencing the choice of the anti-rheumatic agents and on the identification of a temporary window in which the administration of immune-targeted treatment could prevent the progression to life-threatening disease in pediatric patients.

## The rationale for the use of anti-rheumatic agents in pediatric COVID-19

Knowledge of the pathogenesis of severe COVID-19 and MIS-C derives from hypotheses from clinical, epidemiological, pathophysiological observations, and current immunological assumptions. The high variability in the incubation period and the clinical phenotype, as well as the complex biology of the interplay between the infectious agent and the immune system, is under intense investigation [14]. Similarly, the reduced susceptibility to ARDS in children remains to be explained, although differences in the innate and adaptive immune response, together with extra-immunological factors (ACE2 receptor expression and function, baseline pro-inflammatory state) have been proposed to contribute to this phenotypic variability [15]. Data supporting the involvement of the immune system in the pathogenesis of COVID-19 and MIS-C derive also from the analysis of patients with chronic immune-mediated disorders treated with immunosuppressive agents. Indeed, literature data do not show an increased risk of severe COVID-19 in patients with inflammatory bowel disease, autoimmune and anti-inflammatory disorders treated with drugs acting on the immune system [16, 17]. Although some studies suggested that these patients could be protected against the development of severe COVID-19, data are still not univocal [17].

### Pathogenic hypothesis-severe COVID-19

Different studies reported that both innate and adaptive immunity are involved in the process leading to severe COVID-19 and ARDS, and available evidence supports the hypothesis of a two-step pathogenic process. The first stage is featured by viral replication, production of the pathogen-associated molecular pattern (PAMPs) by infected cells, and activation of the innate immune response with consequent tissue injury, followed by the release of the damage-associated molecular pattern (DAMPS), such as the high mobility group box-1 (HMGB-1) protein [14]. In the innate immune response to coronaviruses, toll-like receptor (TLR) activation, type 1 and 3 interferons (IFN I-III) release and the complement cascade play a central role, together with the adaptive immune response (T helper and T cytotoxic cells, antibodies) to favor the clearance of the infectious agent [15, 18]. In the second step, when the infectious process is not adequately controlled, the spreading of the mediators produced in the first phase (cytokines, chemokines, PAMPS, DAMPS) is responsible for the development of an aberrant inflammatory response, associated with pulmonary and systemic organ damage. Cells and mediators of the adaptive immune response contribute in this pathogenic stage by causing an amplification of the inflammatory response and tissue damage, which can be further enhanced in the case of bacterial superinfection [14]. Therefore, as further discussed in this paper, inflammatory involvement is a prominent feature of severe COVID-19, its degree is pivotal to determining disease severity, and therapeutic efforts should be directed to prevent the second phase of the pathogenic process.

### Pathogenic hypothesis-MIS-C

The pathogenesis of MIS-C and its association with SARS-CoV-2 infection still represent a matter of debate. Indeed, although the temporary association between the COVID-19 pandemic and the emergence of MIS-C has been clearly evidenced in Western countries, it has not been confirmed in areas where KD is endemic, opening to the hypothesis that MIS-C could be associated with other infections or environmental changes [14]. Previous studies evidenced that about 40% of the patients test positive for a RT-PCR for SARS-CoV-2 and most of the patients exhibit anti-SARS-CoV-2 antibodies [19]. However, further studies are needed to confirm this pathogenic association and to demonstrate the specificity of RT-PCR and serologic testing for SARS-CoV-2. Following the hypothesis of a pathogenic association between SARS-CoV-2 and MIS-C, the most accredited theories to explain the development of the disease suggest the involvement of both the innate and adaptive response. Delayed IFN response with consequently enhanced viral replication and cytokine release, antibody-mediated tissue damage, and defective T-cell response could contribute to this complex pathogenic and clinical spectrum [15, 20, 21]. Interestingly, Consiglio et al. evidenced some differences in the cytokine profile between MIS-C and KD patients, with higher levels of IL-17 being observed in the KD group [20].

Finally, a role for the gut microbiome in the pathogenesis of MIS-C and severe COVID-19 has been proposed. Indeed, the gut microbiome, connected with the lung immune system in an immunological synapsis defined as the “gut-lung axis”, influences the local and systemic production of a wide variety of cytokines with pro-inflammatory and antiviral activity, including IFN^22^. Moreover, it modulates the function of the immune system through the interaction with TLR, antigen-presenting cells, T helper cells and other molecular targets [22, 23]. Therefore, microbiome differences could contribute to the pathogenesis of ARDS and to determine the heterogeneity of the clinical and immunological factors in COVID-19, thus suggesting that acting on the microbiome could represent a promising adjuvant therapeutic and prophylactic strategies during the COVID-19 pandemic [23, 24]. As some authors hypothesized that the differences in the incidence of KD among different geographic areas could partly depend on different microbiome strains colonization [25], it could be speculated that a similar mechanism could also be involved in the pathogenesis of MIS-C. In this regard, environmental changes related to the COVID-19 pandemic (sedentary lifestyle, dietary variations) could contribute to altering the microbiome composition and, consequently, to the development of an imbalanced immune response [14].

## Anti-rheumatic drugs in COVID-19: an overview

**Fig. 1:**
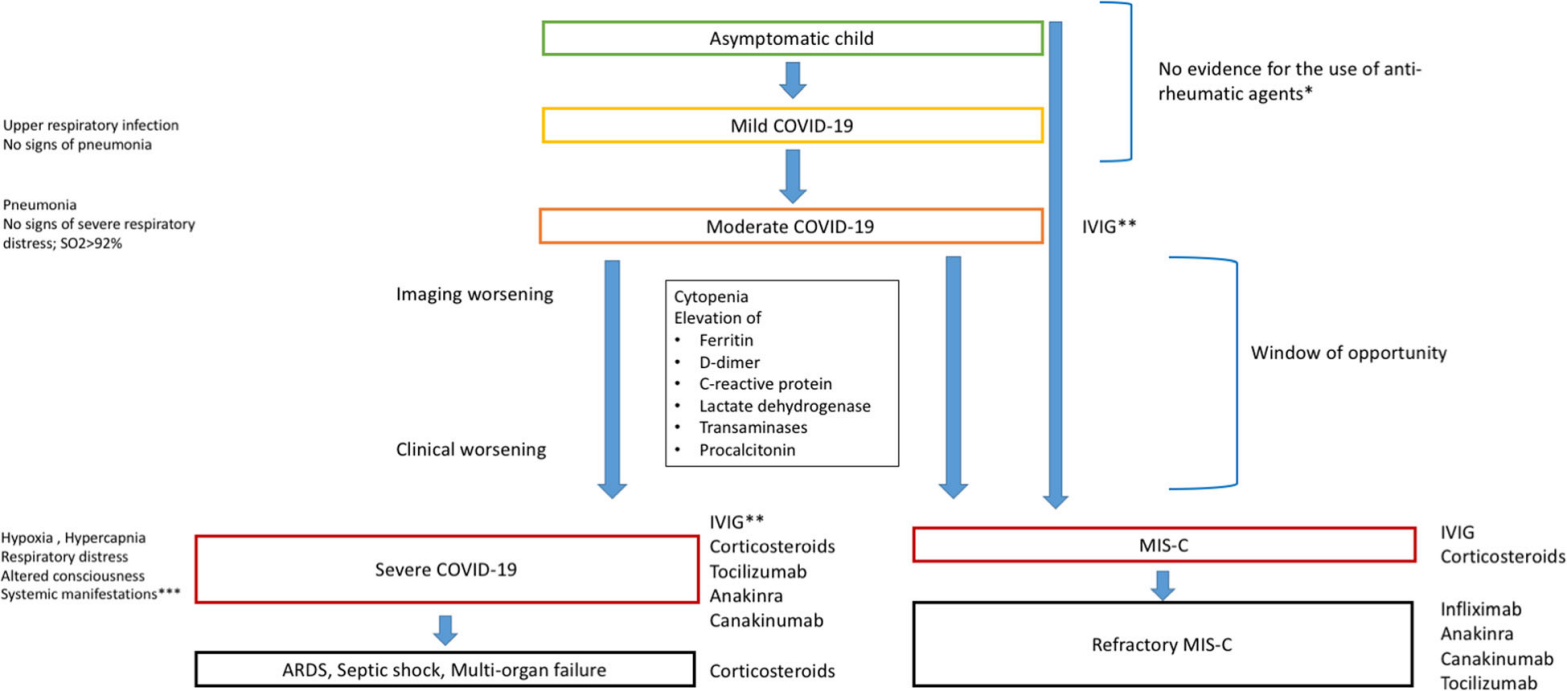
Treatment of COVID-19 in children with a focus on anti-rheumatic agents: current options. The figure summarizes the available anti-rheumatic agents for the treatment of COVID-19, highlighting their potential application according to disease severity. ARDS: acute respiratory distress syndrome; IVIG: intravenous immunoglobulin.; * Not sufficient evidence on the role of hydroxychloroquine and colchicine** Indicated in patients showing seroconversion; ** coagulation disorder, dehydration, cardiac or renal dysfunction

Different therapeutic agents acting on the immune system [26] have been proposed for the treatment of COVID-19 and MIS-C, to modify the disease course (Table 1). However, experience with single drugs remains limited, and the correct timing for their use has yet to be correctly defined and standardized. Ongoing clinical trials will help in providing new insight into the immune therapeutic approach to COVID-19 (available at: clinicaltrials.gov). As the pathogenic and clinical knowledge is in constant update and the role of different drugs (corticosteroids, biologic agents) is rapidly evolving, guidelines could have some important limitations, since they cannot be able to fully represent current evidence. Figure 1 evidences the available therapeutic options with anti-rheumatic drugs according to disease severity.

**Table 1:** Current and potential anti-rheumatic drugs in pediatric COVID-19 and MIS-C

Drug	Mechanism of action	Therapeutic rationale	Experience in pediatric age
**Corticosteroids**	Broad-spectrum anti-inflammatory effect, at a transcriptional and post-transcriptional level. Modulation of the expression of cytokines and adhesion molecules	Anti-inflammatory activity useful in patients with severe and progressive ARDS and MIS-C.	Retrospective cohort studies, case reports, and series RCTs (ongoing)
**Hydroxychloroquine**	Altered glycosylation of the ACE2 receptor, alkalization of the endosomal pH.	Direct antiviral effect through inhibition of viral entry and maturation and immune modulation secondary to the interference with TLR signaling.	Retrospective cohort studies RCTs (ongoing)
**Colchicine**	Inhibition of cytokine synthesis and neutrophil chemotaxis	Reduction of the SARS-CoV-2 related hyperinflammation and prevention of disease worsening	Case reports, and series RCTs (ongoing)
**IVIG**	Reduction of antibody-mediated damage. Immune modulation on multiple antibody-independent pathways, including cytokine modulation and complement downregulation.	Reduction of the antibody-dependent enhancement of cytokine production. Potential utility in case of early seroconversion and in MIS-C.	Case reports, and series RCTs (ongoing)
**Tocilizumab, sarilumab**	Monoclonal anti-IL-6 antibodies	Action on the main mediator of the cytokine storm	Retrospective cohort studies, case reports, and series RCTs (ongoing)
**Anakinra**	IL-1 receptor antagonist	Action on the cytokine initiating the cytokine storm, demonstrated efficacy in patients with sepsis	Case reports, and series RCTs (ongoing)
**Canakinumab**	Monoclonal anti-IL-1 antibody	Action on the cytokine initiating the cytokine storm	No experience RCTs (ongoing)
**Baricitinib, ruxolitinib**	JAK/STAT blockers	Inhibition of viral entry, modulation of the immune response and IFN-dependent signaling	No experience RCTs on ruxolitinib (ongoing)
**Emapalumab**	Monoclonal anti-IFN-γ antibody	Action on the cytokine storm	No experience
**Infliximab**	Monoclonal anti-TNF-α antibody	Action on the cytokine storm, proposed for refractory MIS-C	Case reports and series
**Adalimumab**	Monoclonal anti-TNF-α antibody	Action on the cytokine storm	No experience

*ARDS* acute respiratory distress syndrome; *IFN* interferon; *IL* interleukin; *IVIG* intravenous immunoglobulin; *JAK/STAT* janus kinase–signal transducer and activator of transcription; *MIS-C* multisystem inflammatory syndrome in children; *RCT* randomized-controlled trial; *TNF* tumor necrosis factor

### Corticosteroids

The risk/benefit balance derived from the administration of corticosteroids in patients with COVID-19 remains a subject of debate among experts. Indeed, corticosteroids can interfere with the uncontrolled systemic inflammation featured in COVID-19, but can also impair the viral clearance, thus representing a “double-faced weapon” against the disease [27, 28]. Current guidelines recommend the administration of corticosteroids in patients with rapidly progressing ARDS [7, 12], to control the immune-induced damage. As clinical trials on the use of corticosteroids in COVID-19 show a high heterogeneity for what concerns the molecule, the posology, and the timing of administration, the evidence is still low [29]. Indeed, although different studies evidence that treatment with corticosteroids is associated with reduced mortality in patients with severe disease, the choice of the corticosteroid molecule and the posology remain to be defined. In this regard, the large-scale trial by Horby et al. demonstrated that the administration of dexamethasone (6 mg daily) was associated with a significant reduction of the mortality in patients receiving respiratory support (oxygen alone, mechanical ventilation) [30], while in a recent clinical the use of a higher posology of dexamethasone (20 mg daily for the first 5 days, 10 mg in days 6–10) was followed by a reduced duration of mechanical ventilation [31]. The use of methylprednisolone has been analyzed by different studies, confirming that it reduces mortality in patients with ARDS and, interestingly, that its use in combined therapy with intravenous immunoglobulins (IVIG) could represent a therapeutic option in tocilizumab-refractory patients [32]. Finally, studies performed in patients with severe disease showed that the association between corticosteroids and biologic agents can result in reduced mortality compared to biologic agents alone [33].

Concerning MIS-C, in different case series, the administration of intravenous corticosteroids is reported in nearly half of the patients, resulting in a clinical improvement [8, 34].

### Chloroquine and hydroxychloroquine

The antiviral and immunomodulatory mechanism of action of chloroquine and hydroxychloroquine suggested their use on COVID-19 (including mild and moderate disease) since the first stages of the pandemic. Their action against coronaviruses is mediated by the altered glycosylation of the ACE2 receptor, the alkalization of the endosomal pH, which interferes with virus-endosome fusion, and impairment in the maturation of viral proteins [35]. Additionally, the drugs also act by modulating the immune response and the release of cytokines through different molecular pathways, including action on TLRs [36]. However, clinical trials have not shown univocal results in both adult and pediatric populations. Hydroxychloroquine was ineffective in reducing disease duration in non-hospitalized patients, reducing mortality in the severe disease, and preventing the infection when used as postexposure prophylaxis [37–39]. Additionally, concerns on its safety profile have been recently raised [40, 41]. Indeed, although most of the reported side effects of hydroxychloroquine in COVID-19 are mild and self-resolving (nausea, diarrhea, abdominal discomfort, transient elevation of liver enzymes [40]), its use in hospitalized patients has been associated with increased mortality and necessity of mechanical ventilation in large studies [37, 38]. Finally, other safety concerns derive from the fact that hydroxychloroquine can cause prolonged QT interval and arrhythmias, particularly when it is associated with azithromycin, although severe cardiovascular toxicity has been infrequently reported [42]. Following the results of these studies, international guidelines discouraged the use of hydroxychloroquine in patients with COVID-19, independently from the disease severity. Currently, its administration is allowed only in the setting of clinical trials [12].

### Intravenous immunoglobulins

Treatment with IVIG, which has demonstrated effects in patients with severe sepsis [43], also represents a therapeutic option for adult and pediatric COVID-19 patients [44, 45], given the immunomodulatory and anti-inflammatory properties of IVIG. In particular, in COVID-19, IVIG can act by reducing the antibody-dependent (FcR-mediated) activation of macrophages, which enhances the production of pro-inflammatory cytokines [46]. As a result of this hypothesis, add-on therapy with IVIG, as discussed by Ferro et al., could be beneficial in patients with seroconversion, and consequent antibody-mediated perpetuation of the immune response [47]. In children with MIS-C, especially when it presents with Kawasaki-like clinical features, the use of IVIG is associated with significant clinical benefit [34]. This finds its rationale on the massive involvement of the immune response in MIS-C, as this condition occurs mostly in patients with positive serology for SARS-CoV-2 [9]. Moreover, the potential benefit of administering convalescent plasma and hyperimmune immunoglobulin has been proposed; currently, different trials on this matter are ongoing [48] and, although data from published case series show promising results in hospitalized patients [49, 50], the effect on mortality has to be confirmed by further studies [51].

### Colchicine

The interest in the anti-inflammatory drug colchicine derives from its ability in interfering with the cytokine release and neutrophil chemotaxis [52]. Some retrospective studies suggested a potential role of colchicine in preventing the clinical worsening and hospitalization in COVID-19 patients [53], and other studies found promising results in hospitalized patients [54], but there is a lack of clinical data from large multicentric clinical trials. The good safety profile of colchicine, demonstrated also in pediatric patients suffering from autoinflammatory disorders, encourages the research on the use of this drug in the early stages of COVID-19 [55].

### Anti-IL-6 agents

The central role of IL-6 as a main player in the cytokine storm supported the use of the anti-IL-6 antibody, tocilizumab, initially as an off-label drug and subsequently in clinical trials. Although tocilizumab was the first “targeted-therapy” to be proposed against the aberrant inflammatory response in COVID-19 patients, there are no definitive data on its efficacy [7, 56, 57], and data deriving from recent trials (including the phase III trial COVACTA study) [58] performed in hospitalized patients with severe COVID-19 showed conflicting results on the effect of tocilizumab in reducing mortality [58–60]. However, the non-univocal results in different studies can be explained by the fact that the outcome following the use of biologic agents in COVID-19 can be significantly influenced by the correct timing of administration of the treatment [58]. Furthermore, the efficacy, safety, and the optimal time of administration of tocilizumab and the other anti-IL-6 agent sarilumab are being investigated in other clinical trials.

### Anti-IL-1 agents

Knowledge of the pivotal role of IL-1 in the initiation of the cytokine cascade supported the introduction of the anti-IL-1 drugs, anakinra, and canakinumab, as potential therapeutic strategies in COVID-19. Although the experience with the use of anakinra in patients with infectious diseases is limited, it showed efficacy in patients with severe sepsis [61], given its role in controlling the systemic immune response and consequent organ damage. Moreover, its administration is part of the therapeutic alternatives in other cytokine storm syndromes, such as the macrophage activation syndrome (MAS) [62, 63]. The use of anakinra has shown promising results in adult COVID-19 patients with respiratory distress syndrome [64–66], although its efficacy in patients with mild-to moderate pneumonia needs to be further investigated [67]. Also, the use of anakinra has been reported in pediatric cases of severe COVID-19 and MIS-C [34, 45].

### Other therapeutic targets

The use of the Janus kinase–signal transducer and activator of transcription (JAK/STAT) signaling inhibitors, baricitinib, and ruxolitinib, has been proposed as a potential therapeutic strategy in COVID-19 [68]. This class of drugs not only acts to reduce the inflammatory response and the release of cytokines, as it also targets the adaptor-associated kinase (AAK1), central in clathrin-mediated endocytosis [7, 28, 68, 69]; thus potentially inhibiting viral entrance into the cells. The anti-TNF-α monoclonal antibody adalimumab has been proposed for the treatment of COVID-19 [28]. The rationale of its utilization is the up-regulation of tumor necrosis factor-α (TNF-α) demonstrated in murine models of severe acute respiratory syndrome (SARS), and the elevated levels of the cytokine detected in a subgroup of patients with severe disease and cytokine storm [28, 70]. As infliximab is part of the therapeutic alternatives for refractory KD, it can be considered in patients with MIS-C [71], although there are only limited data on its use [72, 73]. Finally, considering that interferon-γ (IFN-γ) is markedly elevated in the serum of patients with severe COVID-19, researchers have suggested a role for its inhibition in the treatment of the disease. In particular, trials are currently ongoing into the efficacy of the monoclonal anti-IFN-γ antibody emapalumab [74].

## Main perspectives for the improvement of anti-rheumatic treatment in pediatric COVID-19

### Defining the correct timing for the administration of anti-rheumatic drugs

Most of the clinical trials for COVID-19 are directed to the adult population, and further studies are needed to optimize the immune approach in children [75], balancing the effect of therapy with potential side effects derived from immunosuppression. Several efforts have been made to identify the correct temporary window for the use of the drugs acting on the immune response. Currently, the administration of anti-rheumatic agents in the early disease stages is not supported by solid evidence, and there is a major focus on the immune-targeted therapeutic approach to the severe disease manifestations.

The critical point in the management of COVID-19 in childhood is to identify potential candidates for moderate disease and, in parallel, to recognize early those who will progress to severe-critical cases. Indeed, according to the current pathogenic hypothesis, treating patients during the first disease stage could prevent the massive release of proteins from injured tissues, the uncontrolled secretion of pro-inflammatory cytokines, and the systemic spreading of the disease [76, 77]. To this end, many studies have worked to identify biomarkers with specificity in predicting a severe disease course, to evidence the transitional stage from the mild to the severe/critical phase of the disease or predicting the development of MIS-C, and therefore select those patients who should be treated with agents acting on the immune response. This could represent a “window of opportunity” for an early therapeutic intervention to inhibit the rapid progression toward a life-threatening disease. Evidence from published literature suggests that, in pediatric patients, the elevation of c-reactive protein (CRP), procalcitonin, D-dimer, and lactate dehydrogenase (LDH) is associated with the development of severe disease [78]. It is noted that these parameters, associated with the elevation of ferritin and the development of cytopenias, are candidates for predictors of severity in the adult population [2, 79–82].

As the clinical manifestations and the cytokine profile observed in severe-critical COVID-19 and MIS-C in children show common features with MAS, we can derive important propositions in terms of both recognition and treatment from our knowledge of this condition. The diagnostic criteria for pediatric MAS, recently revisited by Ravelli et al., include fever; elevation of ferritin, aspartate aminotransferase, and triglycerides; thrombocytopenia, and a reduction of serum fibrinogen [83]. Moreover, the values of fibrinogen, LDH, ferritin, and the platelet count are among the main predictors of MAS in patients with systemic juvenile idiopathic arthritis (sJIA) [84].

As expected, these laboratory findings significantly overlap with the clinical and laboratory picture of severe-critical COVID-19 and MIS-C. Therefore, these biomarkers should be considered among the proposed prognostic factors associated with disease progression, as potential indicators of the need for immune-targeted treatment. In addition to the above-mentioned biomarkers, as the evolution of chest CT findings is used as a predictor of severe evolution in the general population [85], the role of lung ultrasound must be expanded and standardized in children [86] to obtain an integrated clinical, laboratory, and instrumental assessment of patients. We hope that ongoing clinical trials will help to optimize the accuracy of warning signs and provide useful guidance for therapeutic choices, even in pediatric patients.

### Providing a rationale for the choice of the anti-rheumatic drugs

In patients with clinical, imaging, and biochemical findings predicting clinical worsening, a therapeutic approach based on cytokine blockade could find its “window of opportunity” and prevent progression to severe disease, as proposed for the adult population [47]. There is still no consensus on the choice of a specific anti-cytokine agent, with anti-IL-1 and anti-IL-6 drugs being the most widely used in both severe COVID-19 and MIS-C. To this purpose, important considerations on other cytokine storm syndromes, together with data deriving from specific clinical trials, can help in the decision process.

The interest in the treatment with tocilizumab derives from the ability of IL-6 to enhance, amplify, and maintain the inflammatory response. The efficacy and safety demonstrated by its use in other rheumatologic pediatric diseases (MAS, sJIA, monogenic autoinflammatory disorders, and cytokine release syndrome following the administration of CAR-T cell therapy) [87] additionally support the rationale for this treatment. On the other hand, the recognized efficacy of the anti-IL-1 receptor antagonist anakinra and the anti-IL-1 monoclonal antibody canakinumab in sepsis, monogenic autoinflammatory disorders, idiopathic pericarditis, and sJIA with a prevalence of systemic signs, together with the evidence on the role of IL-1 in severe COVID-19 and MIS-C, suggests their use as a promising alternative [88–92]. Currently, there are only limited studies comparing the efficacy and safety profile of tocilizumab and anti-IL-1 agents in COVID-19. In adults, a statistically significant difference in the outcome of patients treated with anakinra or tocilizumab has not been demonstrated [33, 93]. The experience in children is even more limited, as both MIS-C and severe COVID-19 and ARDS are uncommon, but data on the use of biologic agents are promising [45]. In absence of specific recommendations, the choice of the anti-cytokine agent is generally performed on the basis of the individual experience with the single biologic agent and considering the safety profile in the specific patients. In this regard, the short-acting nature of anakinra, together with the low incidence of infections and other adverse effects in patients treated with this drug [94], are important elements supporting its use in pediatric patients with COVID-19. Also, the adoption of combination regimens, such as the association of biologic agents and corticosteroids or IVIG, is not regulated by specific guidelines, as well as the correct posology for the administration of corticosteroids. Therefore, the knowledge of the pathogenic mechanisms of the disease and the data deriving from the numerous clinical trials performed on COVID-19, together with the experience deriving from the treatment of other rheumatologic conditions (MAS, sJIA, and others) should concur in guiding the therapeutic decisions in daily clinical practice, according to the disease severity in each patient.

Furthermore, we note the potential utility of the dosage of serum cytokines, and in particular IL-1 and IL-6 [95]. When available, performing this test in pediatric patients identified as being at risk of severe disease could help to provide a treatment accurately targeted toward the main mediators involved in pathogenesis in the single patient.

### Improving the choice of the anti-rheumatic drugs in MIS-C

As studies on the pathogenesis of MIS-C have shown a high degree of similarity between this condition and KD, current therapeutic approaches for children with MIS-C are mostly derived from those commonly adopted in KD. In particular, the role of IVIG as first-line therapy is widely accepted, as the utility of high-dose pulse intravenous methylprednisolone [71]. Concerning biologic agents, their administration in MIS-C has to be considered only in refractory cases. As clinical experience is limited, the considerations on the choice of biologic agents for refractory MIS-C are depending on expert opinions, with anakinra and tocilizumab being the most frequently reported drugs, while infliximab has been administered in a lower percentage of patients [34, 73]. Recently, a consensus statement suggested the preferential use of infliximab in children with MIS-C and Kawasaki-like clinical presentation, while there is no univocal indication for the treatment of children with MIS-C and non-specific clinical features [71].

Finally, it is noteworthy that the cytokine profile and the similarities of MAS, pediatric severe COVID-19, and MIS-C may also suggest the potential utility of conventional immunosuppressive agents approved for pediatric MAS. However, the use of those drugs, including cyclosporine, is characterized by a considerable immune impairment, which may significantly impair the viral clearance. Therefore, we suggest not considering such therapeutic strategies for the management of COVID-19 and MIS-C.

## Concluding remarks

Despite the rarity of severe COVID-19 and MIS-C in pediatric patients, clinicians must be prepared to recognize and treat the life-threatening phase of the disease early. Available data on the immune pathogenesis, together with experience gained in the treatment of pathogenic conditions featuring a redundant release of pro-inflammatory cytokines, support the potential utility of therapies targeting the immune system, even in pediatric patients. This work has some limitations deriving from the heterogeneity in the design of the analyzed clinical trials, the continuous evolution of the knowledge in this field and the unmet need to completely clarify the pathogenesis of COVID-19 and MIS-C. However, it provides an updated overview of the current evidence regarding the treatment of these conditions with anti-rheumatic agents and offers interesting research perspectives to improve the therapeutic approach. In particular, we highlight the need to define risk factors associated with disease progression, in order to identify a window of opportunity for the use of therapeutic strategies acting on the immune system to prevent the life-threatening phase of the disease among children. Novel insights into the disease pathogenesis and information from clinical trials will help to define those patients eligible for the treatment with anti-rheumatic drugs. Moreover, as research is rapidly progressing, we hope that in the near future, the choice of an immune-active agent could be made based on the individual clinical evolution and cytokine profile. The final aim of our report is to provoke debate in the scientific community and to stimulate new ideas regarding perspectives on immune responses and the modality and timing of therapeutic strategies acting on the immune system in pediatric patients with severe COVID-19.

## Data Availability

Not applicable.

## Abbreviations

AAK1: Adaptor-associated kinase
ARDS: Acute respiratory distress syndrome
COVID-19: Coronavirus disease 2019
CRP: C-reactive protein
DAMPS: Damage-associated molecular pattern
KD: Kawasaki disease
HMGB-1: High mobility group box-1
IFN: Interferon
IL: Interleukin
IVIG: Intravenous immunoglobulin
JAK/STAT: Janus kinase–signal transducer and activator of transcription
LDH: Lactate dehydrogenase
MAS: Macrophage activation syndrome
MIS-C: Multysistemic inflammatory syndrome in children
MOF: Multi-organ failure
PAMPS: Pathogen-associated molecular pattern
RCT: Randomized-controlled trial
SARS: Severe acute respiratory syndrome
sJIA: Systemic juvenile idiopathic arthritis
TLR: Toll-like
TNF: Tumor necrosis factor
